# Supplementary material for: Re-Evaluating Human Cytomegalovirus Vaccine Design: Prediction of T Cell Epitopes
Source: Vaccines (Basel). 2023 Oct 24;11(11):1629. doi: 10.3390/vaccines11111629 (PMC10674879; doi:10.3390/vaccines11111629)
Supplement: Supplementary file 1 [file vaccines-11-01629-s001.zip › vaccines-2612288-supplementary.pdf]

Table S1. HCMV proteins in each set of the analysis. Column headings indicate set names, number of HCMV proteins in each set, and the rule applied to each set from left to right (see also Figure 1). Shaded rows indicate proteins excluded at each step. The initial exclusion notes the protein and associated measures, and subsequent exclusions are blank. For example, TRL1 (4th row of proteins) was included in the Test Set given no T cell responders, but was excluded from the Identity Set given sequence identity below threshold, and the row is blank in subsequent sets.

* Only the sequence of amino acids 223-484 aligns with US15 in clinical isolates. The original annotation for US15 (CAA35282) in Sylwester et al. (Ref 55) Supplemental Table 1 also contains amino acids 1-222, but this sequence does does not align with any GenBank entry.

** Only amino acids 37-161

† Median immungenicity score significantly lower than that of UL83 (Mann Whitney).

NFL, Not Full Length (n = 12). ASO, AD169-specific ORF (n = 48). COMB, Combined exons (n = 3). DUPL, Duplication (n = 3)

Original Set n = 214			Revised Set n = 148	Test Set n = 109 ≤ 4 CD4 AND ≤ 4 CD8 T cell responders			Identity Set n = 61 ≥ 98% sequence identifiy		Affinity-HLA Set n = 35 High affiity binding to most HLA alleles			Final Set n = 24 High immungenicity	
HCMV Protein	Gene Bank or Swiss Prot ID	Reason for exclusion from Revised Set	HCMV Protein	HCMV Protein	CD8 (#)	CD4 (#)	HCMV Protein	Sequence identity (median %)	HCMV Protein	Peptides with IC50≤50nM (#)	Restricting HLA alleles (#)	HCMV Protein	Immunogenicity score (median) †
IRL14	CAA35296	NFL											
IRS1	CAA35311		IRS1	IRS1	5	13							
J1I	CAA35310	ASO											
TRL1	CAA35449		TRL1	TRL1	0	0	TRL1	92.3					
TRL2	CAA35450	ASO											
TRL3	CAA35451	ASO											
TRL4	CAA35453	ASO											
TRL5	CAA35452	ASO											
TRL6	CAA35454		TRL6	TRL6	0	0	TRL6	98.2	TRL 6	10	7		
TRL7	CAA35455	ASO											
TRL 8	CAA35456	ASO											
TRL9	CAA35457	ASO											
TRL10	CAA35458		TRL10	TRL10	1	3	TRL10	94.7					
TRL11	CAA35459		TRL11	TRL11	0	2	TRL11	96.6					
TRL12	CAA35460		TRL12	TRL12	6	4							

Original Set n = 214			Revised Set n = 148	Test Set n = 109 ≤ 4 CD4 AND ≤ 4 CD8 T cell responders			Identity Set n = 61 ≥ 98% sequence identifiy		Affinity-HLA Set n = 35 High affiity binding to most HLA alleles			Final Set n = 24 High immungenicity	
HCMV Protein	Gene Bank or Swiss Prot ID	Reason for exclusion from Revised Set	HCMV Protein	HCMV Protein	CD8 (#)	CD4 (#)	HCMV Protein	Sequence identity (median %)	HCMV Protein	Peptides with IC50≤50nM (#)	Restricting HLA alleles (#)	HCMV Protein	Immunogenicity score (median) †
TRL13	CAA35461	COMB	TRL13/14	TRL13/14	9	5							
TRL14	CAA35433												
TRS 1	CAA35269	NFL											
UL 1	CAA35434		UL 1	UL 1	0	0	UL1	76.2					
UL 2	CAA35435		UL 2	UL 2	1	1	UL2	93.3					
UL 3	CAA35436	ASO											
UL 4	CAA35437		UL 4	UL 4	1	4	UL4	88.7					
UL 5	CAA35438		UL 5	UL 5	0	0	UL5	97					
UL 6	CAA35439		UL 6	UL 6	0	0	UL6	85.2					
UL 7	CAA35440		UL 7	UL 7	1	0	UL7	87.8					
UL 8	CAA35441	NFL											
UL 9	CAA35442		UL 9	UL 9	0	1	UL9	58.6					
UL 10	CAA35443		UL 10	UL 10	0	0	UL10	95.3					
UL 11	CAA35444		UL 11	UL 11	0	1	UL11	71.7					
UL 12	CAA35446	ASO											
UL 13	CAA35445		UL 13	UL 13	0	3	UL 13	98.1	UL 13	149	20		
UL 14	CAA35447		UL 14	UL 14	1	0	UL 14	99.1	UL 14	70	18		
UL 15	CAA35415	ASO											
UL 16	CAA35448		UL 16	UL 16	1	4	UL 16	98.3	UL 16	81	18		
UL 17	CAA35416		UL 17	UL17	0	5							
UL 18	CAA35417		UL 18	UL 18	1	4	UL 18	97.4					
UL 19	CAA35418		UL 19	UL 19	0	0	UL 19	98	UL 19	50	15		
UL 20	CAA35419		UL 20	UL 20	0	3	UL 20	86.5					
UL 21	CAA35420	ASO											
UL 21.5	P16845		UL 21.5	UL 21.5	1	2	UL21.5	87.5					
UL 22	CAA35421	ASO											
UL 23	CAA35422		UL 23	UL 23	3	1	UL 23	98.9	UL 23	116	20		
UL 24	CAA35423		UL 24	UL 24	1	5							
UL 25	CAA35424		UL 25	UL 25	1	9							

Original Set n = 214			Revised Set n = 148	Test Set n = 109 ≤ 4 CD4 AND ≤ 4 CD8 T cell responders			Identity Set n = 61 ≥ 98% sequence identifiy		Affinity-HLA Set n = 35 High affiity binding to most HLA alleles			Final Set n = 24 High immungenicity	
HCMV Protein	Gene Bank or Swiss Prot ID	Reason for exclusion from Revised Set	HCMV Protein	HCMV Protein	CD8 (#)	CD4 (#)	HCMV Protein	Sequence identity (median %)	HCMV Protein	Peptides with IC50≤50nM (#)	Restricting HLA alleles (#)	HCMV Protein	Immunogenicity score (median) †
UL 26	CAA35425		UL 26	UL 26	2	0	UL 26	98.9	UL 26	44	20		
UL 27	CAA35426		UL 27	UL 27	0	1	UL 27	98.7	UL 27	186	23	UL 27	0.086
UL 28	CAA35427	COMB	UL 28/UL29	UL 28/UL29	10	4							
UL 29	CAA35428												
UL 30	CAA35429		UL 30	UL 30	0	0	UL30	96.7					
UL 31	CAA35430		UL 31	UL 31	0	1	UL 31	99.2	UL31	201	20		
UL 32	CAA35431		UL 32	UL 32	9	13							
UL 33	CAA35432*		UL 33	UL 33	6	1							
UL 34	CAA35393		UL 34	UL 34	5	0							
UL 35	CAA35394		UL 35	UL 35	0	0	UL 35	99.4	UL 35	158	23	UL35	0.013†
UL 36	CAA35395		UL 36	UL 36	4	15							
UL 37	CAA35396		UL 37	UL 37	4	4	UL37	91.2					
UL 38	CAA35397		UL 38	UL 38	3	1	UL 38	98.6	UL 38	118	20		
UL 39	CAA35398	ASO											
UL 40	CAA35399		UL 40	UL 40	3	1	UL40	96.4					
UL 41	CAA35400	ASO											
UL 41a	CAA74073		UL 41a	UL 41a	0	0	UL 41a	98.7	UL 41a	30	11		
UL 42	CAA74074		UL 42	UL 42	0	0	UL42	94.7					
UL 43	CAA74075		UL 43	UL 43	2	4	UL 43	99.1	UL 43	151	21	UL 43	0.041
UL 44	CAA35403		UL 44	UL 44	6	6							
UL 45	CAA35404		UL 45	UL 45	4	3	UL 45	99.5	UL 45	319	26	UL 45	0.077
UL 46	CAA35405		UL 46	UL 46	0	2	UL 46	99.7	UL 46	121	22	UL46	0.020†
UL 47	CAA35406		UL 47	UL 47	2	1	UL 47	99.7	UL 47	332	26	UL 47	0.074
UL 48	CAA35407		UL 48	UL 48	21	15	UL48	99.3	UL48	542	26	UL48	0.081
UL 48.5	YP_081507.1		UL 48.5	UL 48.5	3	2	UL 48.5	98.7	UL 48.5	29	11		
UL 49	CAA35408		UL 49	UL 49	6	4							
UL 50	CAA35409		UL 50	UL 50	2	2	UL 50	99.3	UL 50	66	19		
UL 51	CAA35410		UL 51	UL 51	0	3	UL 51	99.5	UL 51	31	12		
UL 52	CAA35411		UL 52	UL 52	0	9							

Original Set n = 214			Revised Set n = 148	Test Set n = 109 ≤ 4 CD4 AND ≤ 4 CD8 T cell responders			Identity Set n = 61 ≥ 98% sequence identifiy		Affinity-HLA Set n = 35 High affiity binding to most HLA alleles			Final Set n = 24 High immungenicity	
HCMV Protein	Gene Bank or Swiss Prot ID	Reason for exclusion from Revised Set	HCMV Protein	HCMV Protein	CD8 (#)	CD4 (#)	HCMV Protein	Sequence identity (median %)	HCMV Protein	Peptides with IC50≤50nM (#)	Restricting HLA alleles (#)	HCMV Protein	Immunogenicity score (median) †
UL 53	CAA35412		UL 53	UL 53	2	0	UL 53	99.5	UL 53	84	20		
UL 54	CAA35413		UL 54	UL 54	4	1	UL 54	99.5	UL 54	326	25	UL54	0.014†
UL 55	CAA35414		UL 55	UL 55	7	28							
UL 56	CAA35371		UL 56	UL 56	4	0	UL 56	99.4	UL 56	305	26	UL56	0.015†
UL 57	CAA35372		UL 57	UL 57	3	1	UL 57	99.8	UL 57	439	25	UL57	0.051†
UL 58	CAA35373	ASO											
UL 59	CAA35374	ASO											
UL60	CAA35375	ASO											
UL 61	CAA35376	ASO											
UL 62	CAA35377	ASO											
UL 63	CAA35378	ASO											
UL 64	CAA35379	ASO											
UL 65	CAA35380	ASO											
UL 66	CAA35381	ASO											
UL 67	CAA35382	ASO											
UL 68	CAA35383	ASO											
UL 69	CAA35384		UL 69	UL 69	8	6							
UL 70	CAA35386		UL 70	UL 70	0	2	UL 70	99.6	UL 70	341	25	UL 70	0.097
UL 71	CAA35385		UL 71	UL 71	2	2	UL 71	99.2	UL 71	82	16		
UL 72	CAA35387		UL 72	UL 72	0	0	UL 72	99.0	UL 72	104	23	UL 72	0.055
UL 73	CAA35388		UL 73	UL 73	0	0	UL 73	79.0					
UL 74	CAA35389		UL 74	UL 74	0	0	UL 74	80.3					
UL 75	CAA35390		UL 75	UL 75	4	8							
UL 76	CAA35391		UL 76	UL 76	0	1	UL76	97.9					
UL 77	CAA35392		UL 77	UL 77	3	0	UL77	97.8					
UL 78	CAA35351		UL 78	UL 78	3	5							
UL 79	CAA35352		UL 79	UL 79	0	1	UL 79	99.7	UL 79	96	19		
UL 80	CAA35353		UL 80	UL 80	0	1	UL 80	98.2	UL 80	114	22	UL80	-0.059†
UL 80A	CAA35354	NFL											

Original Set n = 214			Revised Set n = 148	Test Set n = 109 ≤ 4 CD4 AND ≤ 4 CD8 T cell responders			Identity Set n = 61 ≥ 98% sequence identifiy		Affinity-HLA Set n = 35 High affiity binding to most HLA alleles			Final Set n = 24 High immungenicity	
HCMV Protein	Gene Bank or Swiss Prot ID	Reason for exclusion from Revised Set	HCMV Protein	HCMV Protein	CD8 (#)	CD4 (#)	HCMV Protein	Sequence identity (median %)	HCMV Protein	Peptides with IC50≤50nM (#)	Restricting HLA alleles (#)	HCMV Protein	Immunogenicity score (median) †
UL 81	CAA35355	ASO											
UL 82	CAA35356		UL 82	UL 82	6	8							
UL 83	CAA35357		UL 83	UL 83	19	24	UL83	99.5	UL83	137	22	UL83	0.076
UL 84	CAA35358		UL 84	UL 84	2	0	UL 84	98.5	UL 84	130	23	UL 84	0.066
UL 85	CAA35359		UL 85	UL 85	1	2	UL 85	99.7	UL 85	105	23	UL85	-0.014†
UL 86	CAA35360		UL 86	UL 86	6	22							
UL 87	CAA35361		UL 87	UL 87	3	4	UL 87	99.6	UL 87	328	23	UL 87	0.045
UL 88	CAA35362		UL 88	UL 88	1	0	UL 88	99.8	UL 88	148	24	UL 88	0.091
UL 89	CAA35363		UL 89	UL 89	0	3	UL 89	99.4	UL 89	242	24	UL 89	0.071
UL 90	CAA35364	ASO											
UL 91	CAA35365		UL 91	UL 91	3	0	UL 91	98.2	UL 91	34	9		
UL 92	CAA35366		UL 92	UL 92	0	0	UL 92	99.5	UL 92	103	24	UL 92	0.043
UL 93	CAA35367		UL 93	UL 93	0	0	UL 93	98.7	UL 93	172	21	UL 93	0.115
UL 94	CAA35368		UL 94	UL 94	8	1							
UL 95	CAA35369		UL 95	UL 95	0	0	UL 95	99.1	UL 95	150	23	UL95	0.004†
UL 96	CAA35370		UL 96	UL 96	0	0	UL 96	98.4	UL 96	12	6		
UL 97	CAA35333		UL 97	UL 97	0	0	UL 97	99.4	UL 97	174	22	UL97	0.011†
UL 98	CAA35334		UL 98	UL 98	2	0	UL 98	99.5	UL 98	224	24	UL98	0.027†
UL 99	CAA35335		UL 99	UL 99	5	18							
UL100	CAA35336		UL100	UL100	3	4	UL100	97.5					
UL101	CAA35337	ASO											
UL102	CAA35338	NFL											
UL103	CAA35339		UL103	UL103	2	0	UL103	99.6	UL103	93	21	UL103	0.041
UL104	CAA35341		UL104	UL104	0	0	UL104	99.7	UL104	170	24	UL104	0.095
UL105	CAA35340		UL105	UL105	7	0							
UL106	CAA35342	ASO											
UL107	CAA35343	ASO											
UL108	CAA35344	ASO											
UL109	CAA35347	ASO											

Original Set n = 214			Revised Set n = 148	Test Set n = 109 ≤ 4 CD4 AND ≤ 4 CD8 T cell responders			Identity Set n = 61 ≥ 98% sequence identifiy		Affinity-HLA Set n = 35 High affiity binding to most HLA alleles			Final Set n = 24 High immungenicity	
HCMV Protein	Gene Bank or Swiss Prot ID	Reason for exclusion from Revised Set	HCMV Protein	HCMV Protein	CD8 (#)	CD4 (#)	HCMV Protein	Sequence identity (median %)	HCMV Protein	Peptides with IC50≤50nM (#)	Restricting HLA alleles (#)	HCMV Protein	Immunogenicity score (median) †
UL110	CAA35348	ASO											
UL111	CAA35349	ASO											
UL111A	CAA35350	NFL											
UL112	P16768	NFL											
UL113	CAA35315		UL113	UL113	2	14							
UL114	CAA35316		UL114	UL114	3	3	UL114	99.6	UL114	79	17		
UL115	P16832		UL115	UL115	0	3	UL115	98.2	UL115	77	19		
UL116	CAA35318		UL116	UL116	4	1	UL116	95.2					
UL117	CAA35319		UL117	UL117	1	0	UL117	99.3	UL117	121	22	UL117	0.047
UL118	CAA35320	COMB	UL118/119	UL118/119	0	1	UL118/119	96.8					
UL119	CAA35321												
UL120	CAA35322		UL120	UL120	2	1	UL120	84.6					
UL121	CAA35323		UL121	UL121	0	0	UL121	95.0					
UL122	P19893		UL122	UL122	12	15							
UL123	CAA35325		UL123	UL123	18	12	UL123	97.2	UL123	115	21	UL123	-0.108†
UL124	CAA35326		UL124	UL124	1	0	UL124	96.1					
UL125	CAA35327	ASO											
UL126	CAA35328	ASO											
UL127	CAA35329	ASO											
UL128	CAA35330	NFL											
UL129	CAA35331	ASO											
UL130	CAA35332		UL130	UL130	1	1	UL130	97.7					
UL131	CAA35294	ASO											
UL132	CAA35295		UL132	UL132	3	0	UL132	93.3					
UL133Tol	AAA85872.1		UL133Tol	UL133Tol	0	0	UL133Tol	96.1					
UL134Tol	AAA85873.1		UL134Tol	UL134Tol	0	1	UL134Tol	94.6					
UL135Tol	AAA85874.1		UL135Tol	UL135Tol	1	2	UL135Tol	97.9					
UL136Tol	AAA85875.1		UL136Tol	UL136Tol	1	0	UL136Tol	97.9					
UL137Tol	AAA85876.1		UL137Tol	UL137Tol	0	0	UL137Tol	94.8					

Original Set<br>n = 214			Revised Set<br>n = 148	Test Set<br>n = 109<br>≤ 4 CD4 AND ≤ 4 CD8 T cell responders			Identity Set<br>n = 61<br>≥ 98% sequence identifiy		Affinity-HLA Set<br>n = 35<br>High affiity binding to most HLA alleles			Final Set<br>n = 24<br>High immungenicity	
**HCMV Protein**	**Gene Bank or Swiss Prot ID**	**Reason for exclusion from Revised Set**	**HCMV Protein**	**HCMV Protein**	**CD8 (#)**	**CD4 (#)**	**HCMV Protein**	**Sequence identity (median %)**	**HCMV Protein**	**Peptides with IC50≤50nM (#)**	**Restricting HLA alleles (#)**	**HCMV Protein**	**Immunogenicity score (median) †**
UL138Tol	AAA85877.1		UL138Tol	UL138Tol	1	1	UL138Tol	98.2	UL138Tol	44	19		
UL139Tol	AAA85878.1		UL139Tol	UL139Tol	0	0	UL139Tol	83.0					
UL140Tol	AAA85879.1	NFL											
UL141Tol	AAA85880.1		UL141Tol	UL141Tol	1	9							
UL142Tol	AAA85881.1		UL142Tol	UL142Tol	0	2	UL142Tol	91.8					
UL143Tol	AAA85882.1	NFL											
UL144Tol	AAA85883.1		UL144Tol	UL144Tol	0	4	UL144Tol	83.0					
UL145Tol	AAA85884.1	NFL											
UL146Tol	AAA85885.1		UL146Tol	UL146Tol	1	5							
UL147Tol	AAA85886.1		UL147Tol	UL147Tol	0	3	UL147Tol	89.9					
UL148Tol	AAA85887.1		UL148Tol	UL148Tol	0	3	UL148Tol	98.7	UL148Tol	122	22	UL148Tol	0.092
UL149 Tol	AAA85890.1		UL149 Tol	UL149 Tol	0	0	UL149	91.0					
UL150 Tol	AAA85891.1	NFL											
UL151 Tow	AAA85892.1	ASO											
UL152 Tow	AAA85894.1	DUPL (UL146)											
UL153 Tow	AAA85895.1	DUPL (TRL13/14)											
UL154 Tow	AAA85896.1	DUPL (TRL12)											
US 1	CAA35312		US 1	US 1	5	2							
US 2	CAA35313		US 2	US 2	2	2	US 2	99.5	US2	88	22	US2	0.092
US 3	CAA35314		US 3	US 3	8	5							
US 4	CAA35271	ASO											
US 5	CAA35272	ASO											
US 6	CAA35273		US 6	US 6	0	2	US6	95.6					
US 7	CAA35274		US 7	US 7	0	1	US7	95.1					
US 8	CAA35275		US 8	US 8	1	2	US8	97.8					
US 9	CAA35276		US 9	US 9	1	1	US 9	98.8	US9	103	19		
US10	CAA35277		US10	US10	2	3	US10	98.4	US10	52	17		
US11	CAA35278		US11	US11	1	0	US11	98.6	US11	105	23	US11	0.087
US12	CAA35279		US12	US12	3	3	US12	98.9	US12	162	21	US12	0.091

Original Set n = 214			Revised Set n = 148	Test Set n = 109 ≤ 4 CD4 AND ≤ 4 CD8 T cell responders			Identity Set n = 61 ≥ 98% sequence identifiy		Affinity-HLA Set n = 35 High affiity binding to most HLA alleles			Final Set n = 24 High immungenicity	
HCMV Protein	Gene Bank or Swiss Prot ID	Reason for exclusion from Revised Set	HCMV Protein	HCMV Protein	CD8 (#)	CD4 (#)	HCMV Protein	Sequence identity (median %)	HCMV Protein	Peptides with IC50≤50nM (#)	Restricting HLA alleles (#)	HCMV Protein	Immunogenicity score (median) †
US13	CAA35280		US13	US13	0	0	US13	99.6	US13	169	21	US13	0.132
US14	CAA35281		US14	US14	2	1	US14	97.4					
US15 *	CAA35282		US15	US15	0	2	US15	99.6	US15	148	21	US15	0.096
US17	CAA35284		US17	US17	0	2	US17	99.7	US17	149	20		
US18	CAA35285		US18	US18	2	5							
US19	CAA35286		US19	US19	1	2	US19	98.3	US19	138	19		
US20	P09724		US20	US20	2	2	US20	99.6	US20	169	22	US20	0.025†
US21	CAA35288		US21	US21	0	1	US21	97.9					
US22	CAA35289		US22	US22	1	7							
US23	CAA35290		US23	US23	2	6							
US24	CAA35291		US24	US24	3	3	US24	99.6	US24	166	24	US24	0.103
US25	CAA35292	ASO											
US26	CAA35293		US26	US26	3	6							
US27	CAA35259		US27	US27	1	1	US27	96.9					
US28	P69332.1		US28	US28	1	0	US28	98.9	US28	208	26	US28	0.067
US29	CAA35261		US29	US29	10	0							
US30	CAA35262		US30	US30	5	1							
US31**	CAA35263		US31	US31	0	1	US31	99.4	US31	44	12		
US32	CAA35264		US32	US32	11	1							
US33	CAA35266	ASO											
US34	CAA35265		US34	US34	0	1	US34	96.3					
US35	CAA35267	ASO											
US36	CAA35268	ASO											
Y9K	P21600	ASO											

Table S2. CD4 or CD8 T cell reactivities to Revised Set HCMV proteins. Panels are sorted by A) participant number or B) number of proteins recognized by total T cells (CD4+CD8). Mean 25 and median 21 proteins were recognized. P, participants.

A)

P	CD4 <1%	CD4 >1%	CD8 <1%	CD8 >1%	Total CD4	Total CD8	Total CD4+CD8
P 1	5	0	16	8	5	24	29
P 2	8	2	2	1	10	3	13
P 3	11	0	2	0	11	2	13
P 4	25	3	15	2	28	17	45
P 5	7	0	1	0	7	1	8
P 6	25	4	8	4	29	12	41
P 7	6	2	8	6	8	14	22
P 8	23	3	5	1	26	6	32
P 9	20	9	12	6	29	18	47
P 10	15	3	4	0	18	4	22
P 11	10	0	2	3	10	5	15
P 12	2	2	4	3	4	7	11
P 13	9	0	8	3	9	11	20
P 14	6	1	7	4	7	11	18
P 15	14	3	3	1	17	4	21
P 16	2	0	3	3	2	6	8
P 17	13	2	8	0	15	8	23
P 18	16	3	10	3	19	13	32
P 19	31	4	19	6	35	25	60
P 20	5	0	6	2	5	8	13
P 21	11	7	15	5	18	20	38
P 22	9	5	3	3	14	6	20
P 23	12	4	6	4	16	10	26
P 24	9	3	4	0	12	4	16
P 25	25	2	16	1	27	17	44
P 26	5	0	2	0	5	2	7
P 27	6	0	4	0	6	4	10
P 28	29	2	23	0	31	23	54
P 29	0	2	20	7	2	27	29
P 30	1	6	6	4	7	10	17
P 31	0	3	5	1	3	6	9
P 32	3	1	0	2	4	2	6
P 33	16	4	26	4	20	30	50

B)

P	CD4 <1%	CD4 >1%	CD8 <1%	CD8 >1%	Total CD4	Total CD8	Total CD4+CD8
P 32	3	1	0	2	4	2	6
P 26	5	0	2	0	5	2	7
P 5	7	0	1	0	7	1	8
P 16	2	0	3	3	2	6	8
P 31	0	3	5	1	3	6	9
P 27	6	0	4	0	6	4	10
P 12	2	2	4	3	4	7	11
P 2	8	2	2	1	10	3	13
P 3	11	0	2	0	11	2	13
P 20	5	0	6	2	5	8	13
P 11	10	0	2	3	10	5	15
P 24	9	3	4	0	12	4	16
P 30	1	6	6	4	7	10	17
P 14	6	1	7	4	7	11	18
P 13	9	0	8	3	9	11	20
P 22	9	5	3	3	14	6	20
P 15	14	3	3	1	17	4	21
P 7	6	2	8	6	8	14	22
P 10	15	3	4	0	18	4	22
P 17	13	2	8	0	15	8	23
P 23	12	4	6	4	16	10	26
P 1	5	0	16	8	5	24	29
P 29	0	2	20	7	2	27	29
P 8	23	3	5	1	26	6	32
P 18	16	3	10	3	19	13	32
P 21	11	7	15	5	18	20	38
P 6	25	4	8	4	29	12	41
P 25	25	2	16	1	27	17	44
P 4	25	3	15	2	28	17	45
P 9	20	9	12	6	29	18	47
P 33	16	4	26	4	20	30	50
P 28	29	2	23	0	31	23	54
P 19	31	4	19	6	35	25	60

Table S3. BLASTP summary of Test Set proteins. Shaded cells indicate proteins excluded from the Test Set in the next step of the algorithm.
(A) UL region proteins; (B) US region proteins

A)

	TRL1	IRS1	TRL12	TRL6	TRL10	TRL11	TRL13/14	UL1	UL2	UL4
Access #	CAA35449	CAA35311	CAA35460	CAA35454	CAA35458	CAA35459	CAA35461	CAA35434	CAA35435	CAA35436
Length (aa)	311	846	416	111	171	234	147 - 302	224	60	149 - 152
Min	76.53%	91.35%	33.72%	97.30%	90.06%	94.04%	41.46%	55.36%	90.00%	79.19%
Max	99.68%	99.76%	99.76%	100.00%	99.42%	99.57%	99.67%	99.55%	98.33%	100.00%
Mean	91.92%	95.13%	48.95%	98.57%	94.80%	96.40%	64.72%	82.54%	94.17%	90.89%
Median	92.28%	95.37%	44.81%	98.20%	94.74%	96.58%	63.97%	76.21%	93.33%	88.67%

	UL5	UL6	UL7	UL9	UL10	UL11	UL13	UL14	UL16	UL17
Access #	CAA35437	CAA35438	CAA35439	CAA35441	CAA35442	CAA35443	CAA35444	CAA35447	CAA35415	CAA35448
Length (aa)	166	280 - 284	222 - 324	228 - 235	258 - 259	275	473	327	230	104
Min	92.77%	77.42%	79.73%	50.21%	84.77%	65.94%	96.62%	98.17%	96.96%	97.12%
Max	99.40%	100.00%	99.55%	99.12%	99.61%	99.64%	99.79%	99.69%	99.57%	99.04%
Mean	96.08%	88.18%	87.43%	66.11%	93.26%	77.80%	98.23%	99.05%	98.34%	98.19%
Median	96.99%	85.21%	87.84%	58.55%	95.29%	71.74%	98.10%	99.08%	98.26%	98.08%

	UL18	UL19	UL20	UL21.5	UL23	UL24	UL25	UL26	UL27	UL28/29
Access #	CAA35416	CAA35417	CAA35418	CAA35419	CAA35420	P16845	CAA35424	CAA35425	CAA35426	CAA35427
Length (aa)	363 - 368	98	340	104-105	284	300 - 358	656	118	608	700
Min	94.29%	96.94%	74.78%	79.61%	97.89%	98.32%	99.09%	98.94%	97.86%	99.00%
Max	99.73%	98.98%	100.00%	99.03%	100.00%	100.00%	99.85%	99.47%	100.00%	99.86%
Mean	97.35%	98.16%	84.34%	88.87%	98.94%	99.35%	99.54%	99.04%	98.76%	99.51%
Median	97.55%	97.96%	86.51%	87.50%	98.94%	99.44%	99.54%	98.94%	98.68%	99.57%

	UL30	UL31	UL32	UL33	UL34	UL35	UL36	UL37	UL38	UL40
Access #	CAA35429	CAA35430	CAA35431	CAA35432*	CAA35393	CAA35394	CAA35395	CAA35396	CAA35397	CAA35399
Length (aa)	121	595	1,048	412	407	640	476	487	331	221
Min	94.21%	98.49%	98.00%	91.03%	98.77%	98.59%	91.99%	88.75%	96.68%	91.86%
Max	99.17%	99.66%	99.81%	99.49%	99.75%	99.84%	99.79%	99.79%	99.70%	99.55%
Mean	96.83%	99.15%	99.12%	94.95%	99.18%	99.35%	98.77%	91.78%	98.58%	96.57%
Median	96.69%	99.16%	99.24%	93.59%	99.26%	99.38%	98.95%	91.17%	98.64%	96.38%

	UL41A	UL42	UL43	UL44	UL45	UL46	UL47	UL48	UL48.5	UL49
**Access #**	CAA74073	CAA74074	CAA74075	CAA35403	CAA35404	CAA35405	CAA35406	CAA35407	YP_081507.	CAA35408
**Length (aa)**	78	78	423	433	906	290	982	2,241	75	570
**Min**	98.72%	92.68%	98.35%	99.31%	98.68%	99.31%	99.19%	98.84%	97.33%	98.25%
**Max**	98.72%	99.20%	99.76%	99.77%	99.89%	99.66%	100.00%	99.55%	98.67%	99.82%
**Mean**	98.72%	94.97%	99.11%	99.59%	99.35%	99.63%	99.64%	99.28%	98.34%	98.95%
**Median**	98.72%	94.74%	99.05%	99.77%	99.45%	99.66%	99.69%	99.29%	98.67%	98.95%

	UL41A	UL42	UL43	UL44	UL45	UL46	UL47	UL48	UL48.5	UL49
**Access #**	CAA74073	CAA74074	CAA74075	CAA35403	CAA35404	CAA35405	CAA35406	CAA35407	YP_081507.	CAA35408
**Length (aa)**	78	78	423	433	906	290	982	2,241	75	570
**Min**	98.72%	92.68%	98.35%	99.31%	98.68%	99.31%	99.19%	98.84%	97.33%	98.25%
**Max**	98.72%	99.20%	99.76%	99.77%	99.89%	99.66%	100.00%	99.55%	98.67%	99.82%
**Mean**	98.72%	94.97%	99.11%	99.59%	99.35%	99.63%	99.64%	99.28%	98.34%	98.95%
**Median**	98.72%	94.74%	99.05%	99.77%	99.45%	99.66%	99.69%	99.29%	98.67%	98.95%

	UL50	UL51	UL52	UL53	UL54	UL55	UL56	UL57	UL69	UL70
**Access #**	CAA35409	CAA35410	CAA35411	CAA35412	CAA35413	CAA35414	CAA35371	CAA35372	CAA35384	CAA35386
**Length (aa)**	397	361	668	375	1,242	906	850	1,235	744 - 748	946
**Min**	98.25%	96.95%	98.20%	97.39%	99.11%	92.00%	98.59%	98.87%	98.39%	99.26%
**Max**	99.75%	100.00%	99.85%	99.73%	100.00%	99.00%	99.88%	99.92%	99.73%	99.89%
**Mean**	99.18%	99.46%	99.34%	99.27%	99.55%	95.85%	99.39%	99.75%	99.05%	99.60%
**Median**	99.25%	99.45%	99.40%	99.47%	99.52%	95.00%	99.41%	99.76%	99.06%	99.58%

	UL71	UL72	UL73	UL74	UL75	UL76	UL77	UL78	UL79	UL80
**Access #**	CAA35385	CAA35387	CAA35388	CAA35389	CAA35390	CAA35391	CAA35392	CAA35351	CAA35352	CAA35353
**Length (aa)**	361	388	138	466	743	325	642	431	295	708
**Min**	96.68%	97.94%	71.74%	73.19%	95.69%	96.62%	96.57%	96.77%	98.98%	97.46%
**Max**	99.72%	99.74%	99.28%	99.79%	99.87%	99.69%	99.38%	100.00%	99.66%	99.86%
**Mean**	99.19%	98.80%	83.37%	83.06%	97.61%	97.95%	97.88%	98.48%	99.49%	98.42%
**Median**	99.17%	98.97%	78.99%	80.30%	96.77%	97.85%	97.82%	98.38%	99.66%	98.17%

	UL82	UL83	UL84	UL85	UL86	UL87	UL88	UL89	UL91	UL92
**Access #**	CAA35356	CAA35357	CAA35358	CAA35359	CAA35360	CAA35361	CAA35362	CAA35363	CAA35365	CAA35366
**Length (aa)**	559	561	586	306	1,370	941	429	674	111	201
**Min**	98.57%	97.68%	97.10%	99.35%	99.05%	97.56%	90.68%	98.66%	95.50%	98.51%
**Max**	99.82%	99.82%	99.83%	99.67%	99.93%	99.89%	100.00%	99.85%	99.10%	99.50%
**Mean**	99.42%	99.49%	98.52%	99.63%	99.66%	99.27%	99.40%	99.37%	97.77%	99.22%

Median	99.64%	99.47%	98.47%	99.67%	99.78%	99.57%	99.77%	99.41%	98.20%	99.50%

	UL93	UL94	UL95	UL96	UL97	UL98	UL99	UL100	UL103	UL104
Access #	CAA35367	CAA35368	CAA35369	CAA35370	CAA35333	CAA35334	CAA35335	CAA35336	CAA35339	CAA35341
Length (aa)	594	345	531	127	707	584	190	372	249	697
Min	97.47%	97.97%	93.93%	97.64%	98.30%	98.97%	90.05%	96.52%	99.20%	98.71%
Max	99.83%	99.71%	99.81%	99.21%	99.86%	99.83%	99.47%	99.73%	99.60%	99.86%
Mean	98.59%	98.61%	98.39%	98.25%	99.43%	99.39%	97.76%	98.12%	99.51%	99.63%
Median	98.65%	98.55%	98.68%	98.43%	99.43%	99.49%	97.89%	97.46%	99.60%	99.71%

	UL105	UL113	UL114	UL115	UL116	UL117	UL118/119	UL120	UL121	UL122
Access #	CAA35340	CAA35315	CAA35316	P16832	CAA35318	CAA35319	CAA35320	CAA35322	CAA35323	P19893
Length (aa)	956	684	250	603	603	424	346	201	180	411
Min	98.12%	98.39%	98.00%	97.48%	91.17%	98.82%	90.06%	77.72%	92.22%	96.84%
Max	99.90%	99.85%	100.00%	100.00%	99.68%	99.76%	99.71%	99.50%	99.44%	100.00%
Mean	99.69%	99.21%	99.46%	98.32%	95.45%	99.20%	95.49%	85.78%	95.13%	99.38%
Median	99.69%	99.27%	99.60%	98.20%	95.18%	99.29%	96.82%	84.58%	95.00%	99.76%

	UL123	UL124	UL130	UL132	UL133	UL134	UL135	UL136	UL137	UL138
Access #	CAA35325	CAA35326	CAA35332	CAA35295	AAA85872.1	AAA85873.1	AAA85874.1	AAA85875.1	AAA85876.1	AAA85877.1
Length (aa)	491	152	214	270	251	175	328	240	96	169
Min	94.09%	90.85%	97.20%	91.48%	89.15%	92.61%	97.26%	95.51%	93.8%	97.04%
Max	99.80%	99.34%	100.00%	99.63%	99.22%	96.02%	98.48%	99.58%	97.9%	99.41%
Mean	97.16%	96.60%	98.63%	94.47%	96.00%	94.53%	97.90%	97.76%	95.2%	98.35%
Median	96.95%	96.05%	98.60%	93.33%	96.12%	94.59%	97.87%	97.92%	94.8%	98.22%

	UL139	UL141	UL142	UL144	UL146	UL147	UL148	UL149
Access #	AAA85878.1	AAA85880.1	AAA85881.1	AAA85883.1	AAA85885.1	AAA85886.1	AAA85887.1	AAA85890.1
Length (aa)	135	338	306	176	117	159	316	122
Min	59.85%	98.22%	80.26%	77.27%	57.26%	86.16%	97.78%	86.29%
Max	99.26%	100.00%	97.06%	100.00%	99.15%	99.37%	99.68%	95.08%
Mean	84.74%	99.01%	91.09%	87.19%	89.63%	90.90%	98.76%	91.33%
Median	82.96%	98.82%	91.83%	82.95%	96.58%	89.94%	98.73%	90.98%

B)

	US1	US2	US3	US6	US7	US8	US9	US10
Access #	CAA35312	CAA35313	CAA35314	CAA35273	CAA35274	CAA35275	CAA35276	CAA35277

**Length (aa)**	156	199	186	182	225	227	247	185
**Min**	98.71%	97.99%	94.62%	78.69%	91.56%	94.71%	95.55%	96.22%
**Max**	99.36%	99.50%	99.46%	99.45%	99.56%	99.56%	99.60%	99.46%
**Mean**	99.25%	99.24%	98.02%	95.39%	95.41%	97.66%	98.82%	98.20%
**Median**	99.36%	99.50%	97.85%	95.63%	95.11%	97.80%	98.79%	98.38%

	**US11**	**US12**	**US13**	**US14**	**US15**	**US16**	**US17**	**US18**
**Access #**	CAA35278	CAA35279	CAA35280	CAA35281	CAA35282	CAA35283	CAA35284	CAA35285
**Length (aa)**	215	281	261	310	262	309	293	274
**Min**	98.14%	98.22%	98.85%	94.67%	98.47%	98.38%	98.29%	99.64%
**Max**	99.53%	99.64%	99.62%	99.68%	100.00%	100.00%	100.00%	100.00%
**Mean**	98.69%	98.95%	99.47%	97.60%	99.49%	99.21%	99.36%	99.67%
**Median**	98.60%	98.93%	99.62%	97.42%	99.62%	99.35%	99.66%	99.64%

	**US19**	**US20**	**US21**	**US22**	**US23**	**US24**	**US26**	**US27**
**Access #**	CAA35286	P09724	CAA35288	CAA35289	CAA35290	CAA35291	CAA35293	CAA35259
**Length (aa)**	240	254	239	576	592	500	603	362
**Min**	95.83%	98.82%	97.53%	99.13%	99.16%	99.00%	98.51%	95.75%
**Max**	100.00%	100.00%	99.58%	99.83%	99.83%	99.80%	99.83%	99.72%
**Mean**	98.22%	99.44%	98.07%	99.48%	99.68%	99.54%	99.22%	97.35%
**Median**	98.33%	99.61%	97.94%	99.48%	99.66%	99.60%	99.17%	96.88%

	**US28**	**US29**	**US30**	**US31**	**US32**	**US34**
**Access #**	P09704	CAA35261	CAA35262	CAA35263	CAA35264	CAA35265
**Length (aa)**	354	462	349	161	183	163
**Min**	97.74%	97.62%	95.13%	98.76%	98.36%	92.02%
**Max**	99.72%	99.57%	99.71%	99.38%	99.45%	99.39%
**Mean**	98.86%	98.79%	97.56%	99.29%	99.06%	96.12%
**Median**	98.87%	98.70%	97.71%	99.38%	98.91%	96.32%
